# The Prevalence of Nomophobia in Medical Undergraduate Students of Central India

**DOI:** 10.7759/cureus.57056

**Published:** 2024-03-27

**Authors:** Mahek R Mohani, Pratik Phansopkar, Nikita H Seth, Pranali S Fokmare

**Affiliations:** 1 Musculoskeletal Physiotherapy, Ravi Nair Physiotherapy College, Datta Meghe Institute of Higher Education and Research, Wardha, IND

**Keywords:** irritability, panic attacks, nomophobia questionnaire, smartphone, nomophobia

## Abstract

Introduction

Nomophobia is an emerging phobia resulting from people's excessive interaction with mobile phones. This phobia is rapidly increasing due to significant technological innovations and widespread acceptance and usage of mobile phones worldwide. Nomophobia is often associated with complications such as panic attacks, irritability, and anxiety. Smartphone usage is particularly high among the younger population, raising concerns as it generates distress and leads to various problems. This study aims to determine the prevalence of nomophobia among undergraduates.

Method

The study utilized the Nomophobia Questionnaire (NMP-Q) with a minimum sample size of 136. A total of 300 Google Forms (Google, Mountain View, California) were circulated, out of which 172 responses were received. A Google Form comprising 20 questions related to smartphone use and nomophobia was designed and distributed to all undergraduate students, who were requested to complete the form. The data based on their responses were subsequently analyzed.

Results

In this study, approximately 31.40% of students disagreed with experiencing panic when running out of credits or hitting monthly data limits. Additionally, 24.42% of students agreed that not having smartphones made them worried, as their family and friends could not contact them. About 16.86% of students strongly disagreed with feeling uneasy because they could not stay updated without their phones. Furthermore, 16.28% strongly agreed that they felt anxious due to the inability to contact their family and friends when not having smartphones.

Conclusion

It can be concluded from this study that nomophobia is present among undergraduate students. The overall usage of smartphones has increased in this population, highlighting the emergence of a serious disorder that warrants attention. Consequently, the usage of smartphones should be reduced through structured training programs, as this proves to be an effective method for enhancing undergraduates' understanding of the prevention and treatment of nomophobia.

## Introduction

Smartphone utilization has surged among today's kids and the younger generation over the last decade, coinciding with an increase in low mental stability within this group. Smartphone ownership has become ubiquitous among youngsters aged 11 and above, and mental health concerns have seen a notable rise among teenagers [1-3]. A study titled "Smartphone Addiction in University Students and Its Implication for Learning" revealed that higher dependency levels were associated with poorer self-control over learning and focus during studying [4,5].

The global health pandemic, COVID-19, prompted individuals to shift their daily routines to isolated locations with the aid of technology [6,7]. Adolescents and young adults aged 15-30 were particularly impacted during the lockdown due to their imprudent reliance on automation, especially those engaged in virtual learning across diverse vocations (42.6 percent) [8]. Nomophobia, an advanced phobia stemming from interactions with mobile mass communication gadgets, particularly cell phones, has emerged as a consequence [9].

Various definitions exist for nomophobia, such as the phobia or distress arising from being cut off from smartphone interaction or without a cell phone [10,11]. Another definition is that the denial of possession of a smartphone, computer, or other digital interpersonal device causes distress or uneasiness, known as nomophobia, resembling a long-term panic disorder termed "social phobia" [12,13].

Regarding nomophobia statistics, a study in the United Kingdom found that 66 percent of people feared losing or being isolated from their phones, with 41 percent owning multiple phones. Women, in particular, exhibited a 70 percent majority in concentration levels and were responsible for higher degrees of worry [14]. Nomophobia, as a legitimate concern, affected millions globally, with those aged 18 to 24 being the most affected. Signs of nomophobia include never shutting off the phone, checking it frequently to see if there are any missed calls or texts, carrying the smartphone around, misusing it, and giving phone conversations priority over in-person conversations [15].

Typical nomophobia traits involve never shutting down the phone, constantly looking for texts and calls that were missed [16], carrying the smartphone everywhere, using it inappropriately, and preceding chances for face-to-face communication above phone calls [5]. Some individuals may experience physical side effects like extreme anxiety, breathlessness, trembling, perspiration, an increased heart rate, and joint pain in the hands, neck, and back when their phone breaks or becomes unusable [15]. Despite the initial purpose of these devices being to provide human comfort, their excessive usage has resulted in various physical and psychological consequences, altering many aspects of people's lives [16]. This study aims to determine the prevalence of nomophobia among students studying medicine in central India. 

## Materials and methods

Method

A cross-sectional study was carried out utilizing the Nomophobia Questionnaire (NMP-Q) [17], with a minimum sample size of 136. 300 Google Forms (Google, Mountain View, California) were distributed, out of which 172 participants responded and were included in the study. A Google Form was designed, consisting of a Nomophobia Questionnaire (NMP-Q) comprising 20 questions that gathered information on smartphone utilization, duration, and intent of smartphone handling. Participants were required to provide their agreement to take part in the research by completing the survey [18]. This form was circulated to all undergraduate students, who were requested to complete it. The data based on the responses from the students were then analyzed. The inclusion criteria were students in the 17-25 years age category, both males and females, willing to sign the consent. Exclusion criteria included students with neurological defects, insomnia, and pain in the shoulder, neck, and hands. Ethical clearance was obtained from the Institutional Ethical Committee (IEC).

Procedure

**Figure 1:**
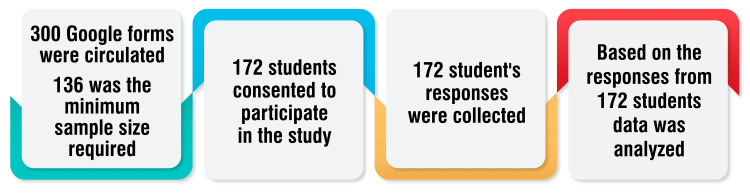
Flowchart

Participants were selected based on the inclusion and exclusion criteria. A Google Form with 20 questions related to smartphone use and nomophobia was circulated to all undergraduate students, who were asked to fill out the form. The data based on the responses were analyzed using the Likert scale. Figure 1 illustrates the methodology of this study in the form of a flowchart.

NMP-Q Using the Likert Scale

Each component was assessed using a seven-point Likert scale, ranging from one (strongly disagree) to seven (strongly agree). The outcome was computed by adding up the answers to each question, producing a nomophobia score that runs from 20 to 140, where a larger number denotes more severe symptoms. A score exceeding 20 but less than 60 indicates mild nomophobia; a total of 60 or more but less than 100 suggests substantial nomophobia, and a score equal to or greater than 100 indicates chronic nomophobia [4]. The questionnaire consisted of twenty items targeting four aspects of NMP: impotence to talk, deprivation of contiguity, inability to acquire knowledge, and giving up liberty. On a seven-point Likert scale, with one denoting "strongly disagree" and seven denoting "strongly agree," responses were evaluated [19].

Sample Size

The sample size was calculated using the estimate proportion formula with absolute precision, considering the prevalence of nomophobia at 6% [20] and the estimation error of 4%. The minimum sample size required was 136.

Statistical analysis

The paired student t-test was utilized to analyze the data with SPSS software version 27 (IRB Inc., Armonk, New York) and GraphPad Prism 7.0 (GraphPad Software, La Jolla, California).

## Results

By distributing 300 Google Forms, the study was successfully conducted among undergraduate students, and the minimum sample size needed was 136, out of which 172 students consented to participate. All tables presented in the appendices represent the statistical analysis of this study.

It was observed that 22% of the students agreed with the notion that they felt uncomfortable without constantly accessing information through their smartphones, while only 8% of the students strongly disagreed (Appendix 1). 21.5% of students expressed annoyance when unable to check information on their smartphones and preferred using their phones whenever desired. There was disagreement among 20% of students regarding feeling nervous when unable to access news, etc., through their smartphones, although 9% agreed to a certain extent. Additionally, 8% of students agreed, while 19% disagreed, that running out of battery scared them.

Based on the responses from the students, it was observed that running out of credits and monthly data limits did not cause panic in around 31% of students (Appendix 2). 23% of students did not express fear of being stranded somewhere without using a smartphone. However, 19.7% of students showed a great inclination to check their phones frequently, either every few minutes or hours. Not having a smartphone made 18% of students feel anxious due to their inability to communicate instantly.

Twenty-four percent of students agreed that the thought of not having their cell phones with them made them worried, as their family and friends couldn't reach them (Appendix 3). Twenty percent of students denied feeling nervous when unable to get texts and calls. Twenty-three percent of students agreed that they felt a bit anxious because they could not keep in touch with their family or friends when not in contact with their phones. Twenty percent of the students accepted that not using phones would break their connection with their near and dear ones.

Thirty-one percent of students disagreed, feeling anxious when cut off from their virtual existence if they didn't have their smartphone with them (Appendix 4). Twenty-seven percent of students disagreed about feeling uncomfortable as a result of their inability to keep up with social media when not using mobile phones. Twenty-four percent denied feeling anxious due to the inability to check their emails when not using phones.

## Discussion

This study employed an observational study design with the aim of determining the prevalence of nomophobia in undergraduate students. The NMPQ was followed, and the data were analyzed. Several similar studies have been conducted worldwide on nomophobia, and according to Notora et al., nomophobia is described as the phobia or fear individuals experience when unable to use mobile phones [21]. It represents a modern type of phobia. A systematic literature review was conducted using the Nomophobia Questionnaire (NMP-Q). It was found that smartphones can have detrimental effects on social, cognitive, and physical well-being [22]. Our study aligns with these findings, demonstrating that not having a smartphone made students feel anxious due to the inability to communicate instantly. Students agreed that they felt a bit worried because they could not keep in touch with their family or friends when not in contact with their phones. This indicates an increase in stress and anxiety levels among students, which could potentially lead to serious problems and impact their social, cognitive, and physical well-being.

Another study was conducted on Bachelor of Medicine and Bachelor of Surgery (MBBS) students, where it was shown that about 19% of them exhibited nomophobia while others did not and were at risk of acquiring nomophobia because 73% of students said they kept their phones nearby when they slept. Additionally, 39% of respondents reported frequently checking their phones for texts and emails. Furthermore, 20% reported that when they don't have their phones with them, they lose focus and become anxious [23]. This study correlates with our research, wherein 56% of students said that they get easily irritated if they can't get updates on smartphones whenever they want to do so. Around 49% of students also agreed that they feel a strong desire to check phones if they last did so a while ago. Forty-eight percent of students felt anxious when they couldn't contact family and friends when away from smartphones. Forty-nine percent of students felt nervous when they couldn't get messages and calls when they did not have a phone with them.

Nomophobia causes many problems; one such study by Alsalameh et al. suggests that smartphone usage has greatly surged, being the main reason for many musculoskeletal issues and musculoskeletal pain [24,25]. The use of smartphones is highest among medical students, leading to pain in the neck and spreading to the lower back, shoulders, and even wrists. Therefore, it is advised to educate students to reduce their dependence or addiction to mobile phones [26].

A study by Jilisha et al. found that as the availability of smartphones rises, so does their usage. The severity of nomophobia and its associated effects among students is underrated in India. Smartphone usage continues to increase across the world; awareness about the potential negative effects, including nomophobia, may grow in India over time, but regarding this, awareness is limited at the present time. Using a semi-structured questionnaire consisting of 20 nomophobia questions, detailed interviews were conducted, revealing average and extreme nomophobia scores. Out of 774 students, 23.5% had high nomophobia scores, with few showing indications of chronic nomophobia. Students also experienced fear and frustration when not using smartphones or when away from their smartphones [27]. This study corresponds with our research, as the students in both studies agreed to experience anxiety when away from smartphone use.

The fact that this disorder significantly affects people's health makes it crucial to organize educational programs in this field for university students. These initiatives could take the form of sessions and consultation sessions on media use to discuss this issue. For the treatment of low rates of nomophobia, other measures, including sports and social programs, are advised, but pharmacological therapy should be employed in more severe cases [28].

A novel approach to treating nomophobia psychologically is called seven magic days. Modeling handling, wherein individuals will be given specific assignments to break their dependence on their phones, a quarantine of seven magic days will be created, during which the participants will be divided into various groups. Numerous initiatives are carried out to educate users about the adverse consequences of using a cell phone [29]. It aims to alter users' actions and thinking patterns so that they become less dependent on their mobile devices. Participants are anticipated to gain a greater understanding of direct interaction with the environment. The goal of the seven magic days is to be accomplished so each individual will only use their phone as necessary and not excessively [30,31]. One limitation of the study was that the method of data collection was through an online survey. However, using the one-to-one survey method could have increased the authenticity of the study. Other limitations of the study were the small sample size and that the study provides information on a specific population only.

## Conclusions

The results of this study indicate that the overall usage of smartphones has increased among undergraduate students, significantly impacting their psychological well-being. Many students experience anxiety when separated from their smartphones, emphasizing the need for regular monitoring of this disorder as it can pose a threat to the lives of young students. Proper planning for prevention, such as engaging students in sports or extracurricular activities, can contribute to a decrease in nomophobia levels.

## Appendices

Appendix 1

**Table 1:** Showing statistical values for the first five questions of the Nomophobia Questionnaire

Questions	Strongly disagree	Disagree	Somewhat disagree	Neither agree nor disagree	Somewhat agree	Agree	Strongly agree
1. I would feel uncomfortable without constant access to information through my smartphones.	14(8.14%)	30(17.44%)	30(17.44%)	18(10.47%)	24(13.95%)	38(22.09%)	18(10.47%)
2. I would be annoyed if I could not look information up on my smartphone when I wanted to do so.	14(8.14%)	23(13.37%)	25(14.53%)	13(7.56%)	37(21.51%)	37(21.51%)	23(13.37%)
3. Being unable to get the news (e.g., happenings, weather, etc.) on my smartphone would make me nervous.	21(12.21%)	34(19.77%)	23(13.37%)	24(13.95%)	30(17.44%)	24(13.95%)	16(9.30%)
4. I would be annoyed if I could not use my smartphone and/or its capabilities when I wanted to do so.	16(9.30%)	26(15.15%)	26(15.12%)	19(11.05%)	32(18.60%)	31(18.02%)	22(12.79%)
5. Running out of battery in my smartphone would scare me.	30(17.44%)	32(18.60%)	28(16.28%)	21(12.21%)	32(18.60%)	14(8.14%)	15(8.72%)

Appendix 2

**Table 2:** Showing statistical values from the 6th to 10th questions of the Nomophobia Questionnaire

Questions	Strongly disagree	Disagree	Somewhat disagree	Neither agree nor disagree	Somewhat agree	Agree	Strongly agree
6. If I were to run out of credits or hit my monthly data limit, I would panic.	25(14.53%)	54(31.40%)	14(8.14%)	17(9.88%)	24(13.95%)	23(13.37%)	15(8.72%)
7. If I did not have a data signal or could not connect to Wi-Fi, then I would constantly check to see if I had a signal or could find a Wi-Fi network.	24(13.95%)	33(19.19%)	20(11.63%)	17(9.88%)	29(16.86%)	31(18.02%)	18(10.47%)
8. If I could not use my smartphone, I would be afraid of getting stranded somewhere.	27(15.70%)	40(23.26%)	24(13.95%)	23(13.37%)	26(15.12%)	18(10.47%)	14(8.14%)
9. If I could not check my smartphone for a while, I would feel a desire to check it.	13(7.56%)	32(18.60%)	23(13.37%)	20(11.63%)	32(18.60%)	34(19.77%)	18(10.47%)
10. If I did not have a smartphone with me, I would feel anxious because I could not instantly communicate with my family and/or friends.	13(7.56%)	31(18.02%)	24(13.95%)	22(12.79%)	30(17.44%)	27(15.70%)	25(14.53%)

Appendix 3

**Table 3:** Shows results of 11th to 15th questions of the Nomophobia Questionnaire

Questions	Strongly disagree	Disagree	Somewhat disagree	Neither agree nor disagree	Somewhat agree	Agree	Strongly agree
11. If I did not have my smartphone with me, I would be worried because my family and/ or friends could not reach me.	9(5.23%)	22(12.79%)	17(9.88%)	13(7.56%)	35(20.35%)	42(24.42%)	34(19.77%)
12. If I did not have a smartphone with me, I would feel nervous because I would not be able to receive text messages and calls.	15(8.72%)	35(20.35%)	19(11.05%)	19(11.05%)	30(17.44%)	31(18.02%)	23(13.37%)
13. If I did not have my smartphone with me, I would be anxious because I could not keep in touch with my family and/or friends.	13(7.56%)	27(15.70%)	11(6.40%)	16(9.30%)	38(22.09%)	39(22.67%)	28(16.28%)
14. If I did not have my smartphone with me, I would be nervous because I could not know if someone had tried to get a hold of me.	16(9.30%)	32(18.60%)	17(9.88%)	25(14.53%)	34(19.77%)	26(15.12%)	22(12.79%)
15. If I did not have my smartphone with me, I would feel anxious because my constant connection to my family and friends would be broken.	12(6.98%)	28(16.28%)	18(10.47%)	20(11.63%)	36(20.93%)	33(19.19%)	25(14.53%)

Appendix 4

**Table 4:** Showing results for 16th to 20th questions of the Nomophobia Questionnaire

Questions	Strongly disagree	Disagree	Somewhat disagree	Neither agree nor disagree	Somewhat agree	Agree	Strongly agree
16. If I did not have my smartphone with me, I would be nervous because I would be disconnected from my online identity.	30(17.44%)	53(30.81%)	15(8.72%)	18(10.47%)	25(14.53%)	18(10.47%)	13(7.56%)
17. If I did not have my smartphone with me, I would be uncomfortable because I could not stay up-to-date with social media and online networks.	29(16.86%)	46(26.74%)	22(12.79%)	22(12.79%)	18(10.47%)	20(11.63%)	15(8.72%)
18. If I did not have any smartphone with me, I would feel awkward because I could not check my notifications for updates from my connections and online networks.	25(14.53%)	46(26.74%)	22(12.79%)	25(14.53%)	18(10.47%)	22(12.79%)	14(8.14%)
19. If I did not have any smartphone with me, I would feel anxious because I could not check my email messages.	27(15.70%)	42(24.42%)	21(12.21%)	23(13.37%)	17(9.88%)	24(13.95%)	18(10.47%)
20. If I did not have any smartphone with me, I would feel weird because I would not know what to do.	23(13.37%)	45(26.16%)	19(11.05%)	28(16.28%)	16(9.30%)	23(13.37%)	18(10.47%)
